# Enteric Nervous System Remodeling in a Rat Model of Spinal Cord Injury: A Pilot Study

**DOI:** 10.1089/neur.2020.0041

**Published:** 2020-10-22

**Authors:** Chloë Lefèvre, Anne Bessard, Philippe Aubert, Charles Joussain, François Giuliano, Delphine Behr-Roussel, Marie-Aimée Perrouin-Verbe, Brigitte Perrouin-Verbe, Charlène Brochard, Michel Neunlist

**Affiliations:** ^1^ UMR Inserm 1235, Research Unit, The Enteric Nervous System in Gut and Brain Diseases (TENS), University of Nantes, Nantes, France.; ^2^ Neurological Physical and Rehabilitation Medicine Department, University Hospital of Nantes, Nantes, France.; ^3^ UMR Inserm 1179, Research Unit, Neuromuscular Disability, Physiopathology, Biotherapy, and Applied Pharmacology (END-ICAP), University of Versailles-St-Quentin-en-Yvelines, Montigny-le-Bretonneux, France.; ^4^ Pelvipharm, University of Versailles-St-Quentin-en-Yvelines, Montigny-le-Bretonneux, France.; ^5^ Urology Unit, University Hospital of Nantes, Nantes, France.; ^6^ Digestive Physiology Unit, University Hospital of Rennes, Rennes, France.

**Keywords:** digestive disorders, enteric neurons, neuromuscular response, spinal cord injury

## Abstract

The physiopathology of digestive disorders in patients with spinal cord injury (SCI) remains largely unknown, particularly the involvement of the enteric nervous system (ENS). We aimed in a rat model of chronic thoracic SCI to characterize (1) changes in the neurochemical coding of enteric neurons and their putative consequences upon neuromuscular response, and (2) the inflammatory response of the colon. *Ex vivo* motility of proximal and distal colon segments of SCI and control (CT) rats were studied in an organ chamber in response to electrical field stimulation (EFS) and bethanechol. Immunohistochemical analysis of proximal and distal segments was performed using antibodies again Hu, neuronal nitric oxide synthase, (nNOS), and choline acetyltransferase. Colonic content of acetylcholine and acetylcholinesterase was measured; messenger RNA (mRNA) expression of inflammatory cytokines was measured using reverse transcription quantitative polymerase chain reaction (RT-qPCR) approaches. Compared with the CT rats, the contractile response to bethanechol was significantly decreased in the proximal colon of SCI rats but not in the distal colon. The proportion of nNOS immunoreactive (IR) neurons was significantly reduced in the proximal but not distal colon of SCI rats. No change in proportion of choline acetyltransferase (ChAT)-IR was reported; the tissue concentration of acetylcholine was significantly decreased in the proximal colon of SCI rats. The expression of tumor necrosis factor alpha (TNF-α) and intercellular adhesion molecule-1 (ICAM-1) was significantly reduced in the proximal and distal colon of SCI rats. This study demonstrates that functional motor and enteric neuroplastic changes affect preferentially the proximal colon compared with the distal colon. The underlying mechanisms and factors responsible for these changes remain to be discovered.

## Introduction

Spinal Cord Injury (SCI) causes multiple functional impairments. The severity and the extent of the deficiencies depend on several factors including the level of injury, the completeness of the injury, and whether or not it is flaccid or spastic.^1–3^ Bowel dysfunction, also known as neurogenic bowel, is one of the most prevalent comorbidities associated with SCI.^4^ Symptoms of bowel dysfunction such as constipation, dyschezia, or fecal incontinence have a significant impact on the quality of life of the patient.^5^ In particular, more than one-third of patients with SCI consider digestive disorders to be a more significant disability than walking loss.^6^ Consistently, the recovery of digestive functions is one of the most frequent wishes among patients with SCI.^5,7–11^ In contrast to well-established treatments for urological disorders, efficient treatments for bowel dysfunction are still lacking for patients with SCI. Such lack of results is due in large part to the still largely unknown pathophysiological basis responsible for GI dysfunction in patients with SCI.

Several mechanisms have been proposed to explain digestive disorders in patients with SCI. In physiological conditions, bowel function is the result of coordinated actions between the sympathetic, parasympathetic, and enteric nervous systems (ENS). In particular, the colon receives sympathetic innervation from the hypogastric nerve (spinal level T10–L2) and parasympathetic innervation from the pelvic nerve (spinal level S2–4). Damage of the supraspinal regulation of somatic and autonomic circuitry of the spinal cord was long thought to explain bowel dysfunction in patients with SCI.^2,3,12^ However, the involvement of the ENS in these dysfunctions has been largely overlooked. Indeed, the ENS is an integrative neuronal network comprising more than 100 million neurons and 400 million enteric glial cells, which are distributed along the digestive tract and organized into two major ganglionated plexi—the myenteric plexus (or Auerbach's plexus) and the submucosal plexus (or Meissner's plexus).^13^ Neurons of the myenteric plexus control the motor activity of the gut, such as propulsive movement of colonic contents, even independently of the central nervous system.

As human studies aimed at characterizing ENS in patients with SCI are difficult to design for evident ethical reasons, recent scarce studies have characterized changes in the ENS in animal models of SCI and their impact on gut functions. In particular, preliminary studies have shown alterations of the ENS and bowel function after SCI, such as alteration of gastric emptying and decrease in marked nNOS neurons in the small intestine,^14^ alteration of acetylcholine-mediated contractile response after acute injury,^15^ decrease in the number of enteric neurons, and alterations of colonic motility *in vivo* after chronic high T3 injury.^16^ However, to our knowledge, no study has currently characterized the ENS remodeling in the colon and its impact upon neuromuscular transmission in a model of chronic or semi-chronic low thoracic lesion (T8–12). However, this model allows direct lesion of the thoracolumbal sympathetic center and is relevant for the analysis of long-term modification in the ENS and its functional impact as these lesions are the most frequent in patients with SCI.^17,18^

Therefore, we used a rat model of chronic thoracic SCI to (1) characterize ENS remodeling in the proximal as well as distal colon, (2) determine its *ex vivo* consequences upon neuromuscular contractile response, and (3) characterize the inflammatory response of the colon.

## Methods

### Animals

Female Sprague-Dawley rats (Janvier Laboratories, Le Genest Saint Isle, France), 9 weeks of age and weighting 280 g were housed for 6–7 days in their definitive animal facility, in a controlled environment with free access to food and water before experiments. The experiments were carried out in strict accordance with the European Communities Council Directives 2010/63/UE on the use of laboratory animal and care regulation in force in France (Ministry of Agriculture, Authorization Agreement No. A78-322-3, December 2013 and B78-423-1, July 2017) on a total of nine control (CT) rats and nine spinalized rats, 21 days after they had undergone a T8–9 spinal cord transection.

### Spinalization

The rats were spinalized as described^19^ by the Pelvipharm company and the Inserm research unit U1179 (Neuromuscular Disability: Physiopathology, Biotherapy, and Applied Pharmacology [END-ICAP]; Faculty of Medicine of the University of Versailles-St-Quentin-en-Yvelines, Montigny-Le-Bretonneux, France). Rats were anesthetized with isoflurane (1.2%; Centravet SA, Plancoët, France) and body temperature was maintained at 37°C using a heating pad. A laminectomy was then performed to expose the spinal cord and a segment (T9) was transected with fine scissors under local anesthesia (0.2 mL lidocaine; Centravet SA). A sterile gelform sponge (Gelita-Spon, Gelita Medical BV, Amsterdam, The Netherlands) was inserted between cut ends of the spinal cord to ensure completeness of the section, then the overlying muscles and skin were sutured. The bladder was manually emptied by Credé's maneuver 3 times a day until the rat recovered efficient micturition reflex. Antibiotic prophylaxis consisted of: (1) cefovecin (20 mg/kg; Centravet SA) subcutaneous injection after spinal cord section, (2) oral enrofloxacin (20 mg/kg/day; Centravet SA) during the first and third week post-section, and (3) oral sulfamethoxazole/trimethoprim (50/10 mg/kg/day; Centravet SA) during the second week post-section.

Twenty-one days after spinal cord transection, animals were anesthetized with isoflurane and sacrificed by cervical dislocation. The colon was immediately placed in cold oxygenated (5% CO_2_ and 95% O_2_) Krebs solution containing (in mM) 117.0 NaCl, 4.7 KCl, 1.2 MgCl_2_, 1.2 NaH_2_PO_4_, 25.0 NaHCO_3_, 2.5 CaCl_2_, and 11.0 glucose.

### Evaluation of colonic motility during ENS stimulation

The samples placed in cold oxygenated Krebs solution were transferred to the laboratory (Inserm research unit U1235, TENS, University of Nantes, Nantes, France). Segments of distal and proximal colon were placed in the longitudinal direction in an organ chamber (Radnoti, California, USA) with 15 mL of Krebs solution at 37°C, continuously bubbled with 95% O_2_ and 5% CO_2_. The contractile response of colonic segments was continuously recorded using isometric force transducers (No. TRI202PAD, Panlab, Cornellã, Spain) coupled to a computer equipped with the PowerLab 8/30 System and Labchart data analysis software (AD Instruments, Spechbach, Germany). Segments were stretched with a pre-load of 1 gram, which was maintained during an equilibration period of 60 min.

Then, the segments were subjected to electrical field stimulation (EFS) using an STG 4008 MCS electrical stimulator (Multi Channel Systems, Reutlingen, Germany) to stimulate the ENS. EFS parameters were as follows: train duration, 10 sec; pulse frequency, 20 Hz; pulse duration, 400 μsec; and pulse amplitude, 11 V. This procedure was repeated 3 times spaced for 10 min.

Then, 15 μL of the following drugs were added in the baths and after a 30-min incubation period the same EFS stimulation protocol was repeated: (1) nitric oxide synthase synthase (NOS) inhibitor, *N*-nitro-L-arginine methyl ester (L-NAME, 50 mM, Sigma); and (2) atropine (10^6^ M, Sigma), an antagonist of cholinergic muscarinic receptors. Contractile activity was evaluated by measuring the area under the curve (AUC). Spontaneous contractile activity was evaluated by measuring AUC during 2 min before the first EFS. The EFS-induced response was evaluated by measuring the AUC during the EFS period (EFS 10 sec) and the AUC 1 min after stimulation (EFS 60 sec), in basal, L-NAME, and L-NAME+atropine conditions. For each segment, the nitrergic component of the response to EFS (10 sec or 60 sec) was calculated by the formula: (AUC EFS L-NAME – AUC EFS control)/AUC EFS control and was named L-NAME sensitive AUC. For each segment, the cholinergic component of the EFS response (10 sec or 60 sec) was calculated by the formula: (AUC EFS L-NAME+atropine – AUC EFS L-NAME)/AUC EFS L-NAME and was named Atropine-sensitive AUC.

Concentration-response effects of bethanechol were analyzed by performing a Friedman test followed by a Dunn test. Concentrations for which bethanechol significantly modified the contractility of the tissue compared with the control situation are indicated by # (CT group) or Φ (SCI group). The effects of spinalization were statistically compared with their respective CT groups by performing a two-way analysis of variance (ANOVA) repeated measure; when the difference was significant between the groups, a Bonferroni test was performed to identify the concentrations at which the effects were significantly different (indicated by *). Data were normalized to tissue weight. A value of *p* ≤ 0.05 was considered significant.

### Immunofluorescence staining

Samples of proximal colon (1 cm adjacent to the cecum) and distal colon (1 cm, directly above the segment used in motility studies) were fixed in 0.1 M phosphate-buffered saline (PBS) containing 4% paraformaldehyde (PFA) for 3 h at room temperature. The circular muscle and the mucosa were removed under a dissection microscope to expose the myenteric plexus. The myenteric plexus was then permeabilized for 3 h at room temperature with PBS, 0.1% sodium azide, 4% horse serum, and 0.5% Triton X-100. Tissue was then incubated with the following primary antibodies for 24 h: goat anti-choline acetyltransferase (ChAT; 1:200; Millipore, Billerica, MA, USA), rabbit anti-neuronal NOS (nNOS; 1:1000; Alexis Laboratories, San Diego, CA, USA), and mouse anti-HuC/HuD (1:200, Invitrogen). The preparation was then washed with PBS and incubated for 3 h with the appropriate secondary antibodies: anti-goat Cy3 (carboxymethylindocyanine; 1:500; Jackson ImmunoResearch, Suffolk, UK), anti-rabbit Cy5 (7-amino-4-indodicarbocyanin; 1:500, Jackson ImmunoResearch), and anti-mouse fluorescein isothiocyanate (FITC; 1:500; Jackson ImmunoResearch). After washes with PBS, specimens were viewed and pictures were acquired with a fluorescent microscope (AxioZoom.V16; Zeiss, Marly le Roi, France) associated with Zen 2012 software (Zeiss) and fitted with adequate filter cubes. Finally, pictures were analyzed with Image J software.

### Myenteric ganglion and neurochemical phenotype analysis

Myenteric ganglia were defined under the microscope as entities containing Hu-IR cells separated by a gap clearly distinguishable (about the size of one neuron or even smaller). Structures not clearly identified as ganglia were not analyzed. The numbers of Hu-, ChAT- and nNOS-immunoreactive (IR) myenteric neurons were counted (at least 20 ganglia per condition). To determine the neurochemical phenotype, data were expressed as the number of myenteric neurons per ganglion and as a percentage of ChAT-IR or nNOS-IR neurons normalized to the total number of Hu-IR neurons per ganglion.

### Acetylcholine and acetylcholinesterase assay

Samples of distal colon placed in radioimmunoprecipitation assay (RIPA) buffer (Millipore) and frozen at −80°C were used. Those whole-mount samples were lysed using a Precellys 24-tissue homogenizer (Bertin Technologies, France) and sonication followed using a Vibracell 75186 device (Sonics, Newton, CT, USA). Total protein was quantified using a Bicinchoninic Acid Protein Assay Kit (Thermofisher, USA). Acetylcholine concentration was then determined in tissue homogenates, prepared and dilued at 1 μg/μL (protein), with an Amplex Red Acetylcholine/Acetylcholinesterase Assay Kit (Invitrogen).

### Quantitative PCR analysis

The samples of colonic tissues placed in RA1 buffer were used. Total RNA were extracted from these samples using a NucleoSpin Triprep Kit (Macherey-Nagel, Hoerdt, France, Cat.# 740966) according to the manufacturer's instructions. Once μg purified, messenger RNA (mRNA) was denatured and processed for reverse transcription using Superscript III reverse transcriptase (Thermo Fisher Scientific, Saint-Herblain, France, Cat.#18080044). PCR amplifications were performed using the Absolute Blue SYBR Green Fluorescein Kit (Roche Molecular Biochemicals, Meylan, France, Cat.# AB4166B) and run on a StepOnePlus system (Life Technologies, Cat.# 4376600). The following primers were used:

- TNF-α # NM_012675.3, forward: 5′-GAGGAGAAGTTCCCAAATGGGCT-3′ ; reverse: 5′-TTGGTGGTTTGCTACAGACGTG-3′
- interleukin (IL)-1β # NM_031512.2, forward: 5′-CAGCTTTCGACAGTGAGGAGA -3′ ; reverse: 5′-TTGTCGAGATGCTGCTGTGA-3′
- IL-7 # NM_013110.2, forward: 5′-CTGGATGCCTCCTGGTCAAA-3′ ; reverse: 5′-TGCAGATGACAG GGTTGCTT-3′
- IL-10 # NM_012854.2, forward: 5′-TGCGACGCTGTCATCGATTT-3′ ; reverse: 5′-GTAGATGCCGGGTGGTTCAA -3′
- IFNγ # NM_138880.2, forward: 5′-GCAAAAGGACGGTAACACGA -3′ ; reverse: 5′-TTGTTCACCTCGAACTTGGC-3′
- intercellular adhesion molecule (ICAM)-1 # NM_012967.1, forward: 5′-CGGACTTTCGATCTTCCGACTA-3′ ; reverse: 5′-TTTGTGCTCTCCAGGGTCAG-3′

### Statistical analysis

Statistical analysis was performed using GraphPad Prism V6 software (GraphPad Software, La Jolla, CA, USA). Data were expressed in medians (extremes). Comparisons between CT and SCI rats were performed using non-parametric Mann-Whitney test or ANOVA. A value of *p* ≤ 0.05 was considered significant.

## Results

SCI and CT rats were comparable for age and weight. No deaths occurred during surgery or follow-up.

### Impact of spinal cord injury on neuromuscular transmission

In this part of the study we aimed to study *ex vivo* the induced contractile response during ENS stimulation and to identify the neuromediators potentially involved.

**FIG. 1.**
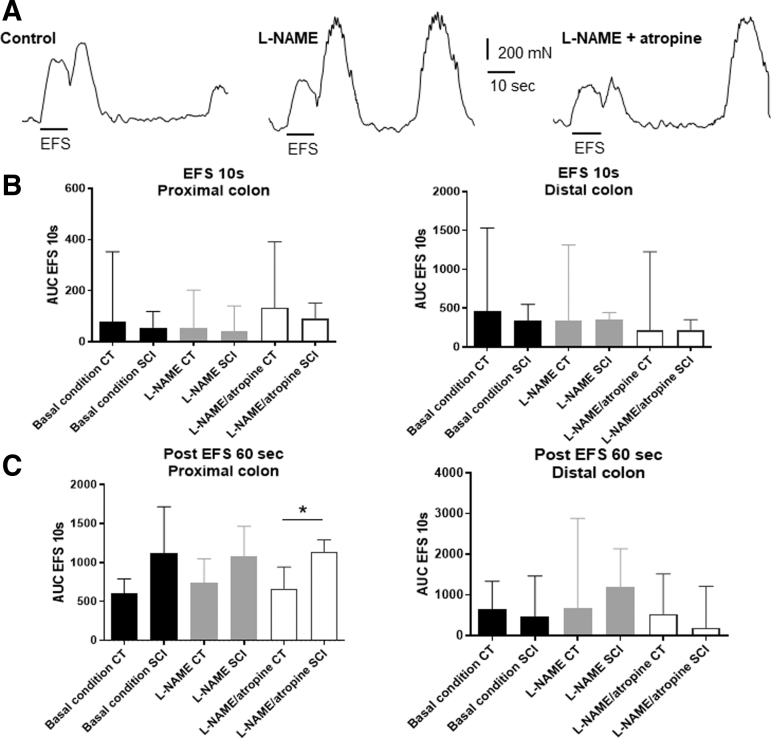
Impact of spinal cord injury (SCI) on neuromuscular transmission assessed *ex vivo*. Proximal and distal colonic longitudinal muscle segments were stimulated by electrical field stimulation (EFS). The area under the curve (AUC) of EFS-induced contractile response was analyzed in absence or in presence of *N*-nitro-L-arginine methyl ester (L-NAME) or atropine (examples, **A**). In basal condition and with drugs, the EFS 10 sec-response was comparable between SCI and control (CT) rats in proximal (basal *p* = 0.30; L-NAME *p* = 0.30; L-NAME+atropine *p* = 0.41) and distal colon (basal *p* = 0,41; L-NAME *p* = 0.66; L-NAME+atropine *p* = 0.80) **(B)**. Post-EFS responses (60 sec post-stimulation) were comparable between the two groups, in proximal and distal colon segments in basal condition (*p* = 0.80 distal colon; *p* = 0.15 proximal colon) and after addition of L-NAME (*p* = 0.53 distal colon; *p* = 0.41 proximal colon). In distal colon only, EFS 60 sec was similar between the two groups in L-NAME+atropine condition (*p* = 0.41). However, in the proximal colon, EFS 60 sec was lower in the SCI group (*p* = 0.03) in L-NAME+atropine condition **(C)**.

EFS-induced contractile response were sequentially analyzed in basal condition and after addition of L-NAME and atropine to determine the contribution of nitrergic and cholinergic components, respectively, in EFS-induced response (Fig. 1A). Under basal condition and with drugs, the area under the curve (AUC) of the response induced during EFS was comparable between SCI and CT rats in proximal (basal: *p* = 0.30; L-NAME: *p* = 0.30; L-NAME+atropine: *p* = 0.41) and distal colon (basal: *p* = 0.41; L-NAME: *p* = 0.66; L-NAME+atropine: *p* = 0.80) (Fig. 1B). The AUC of post-EFS responses (i.e., 60 sec post-EFS) were also comparable between the two groups, in proximal and distal colon segments in basal condition (distal colon: *p* = 0.80; proximal colon: *p* = 0.15) and after addition of L-NAME (distal colon: *p* = 0.53; proximal colon: *p* = 0.41). In distal colon, post-EFS response was similar between the two groups in L-NAME+atropine condition (*p* = 0.41). However, in the proximal colon, post-EFS response was lower in the SCI group in L-NAME+atropine condition (Fig. 1C) (*p* = 0.03).

**FIG. 2.**
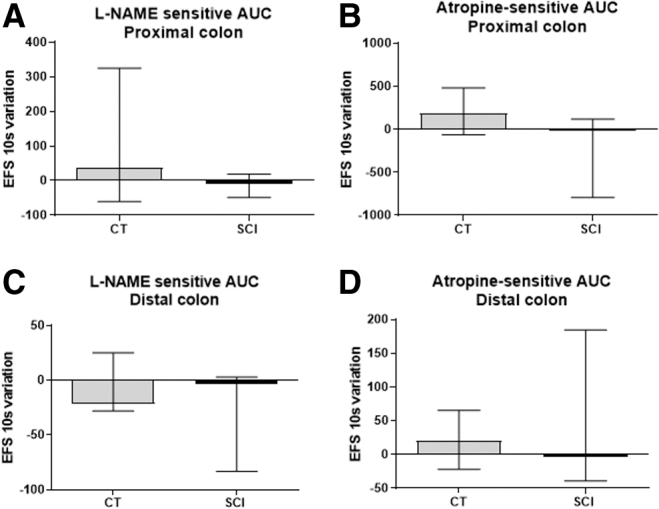
Impact of spinal cord injury (SCI) on neuromuscular transmission assessed *ex vivo*. Proximal and distal colonic longitudinal muscle segments were stimulated by electrical field stimulation (EFS). The area under the curve (AUC) of EFS-induced contractile response was analyzed in absence or in presence of *N*-nitro-L-arginine methyl ester (L-NAME) or atropine. The amplitude of L-NAME-sensitive AUC was similar in the SCI group compared with control (CT) rats in proximal **(A)** (*p* = 0.41) and distal colon **(C)** (*p* = 0.66). The amplitude of the atropine-sensitive AUC tended to be decreased only in the proximal colon in the SCI group **(B)** (*p* = 0.09) but not in the distal colon **(D)** (*p* = 0.53).

In the proximal colon, the amplitude of L-NAME-sensitive AUC was comparable between the two groups (*p* = 0.41) (Fig. 2A) and the amplitude of the atropine-sensitive AUC tended to be decreased (*p* = 0.09) in SCI rats compared with CT (Fig. 2B). In the distal colon of SCI rats, the amplitude of L-NAME sensitive AUC (Fig. 2C) and atropine-sensitive AUC (Fig. 2D) were similar compared with CT (*p* = 0.67 and *p* = 0.53, respectively).

### Impact of SCI on neurochemical phenotype of myenteric neurons

**FIG. 3.**
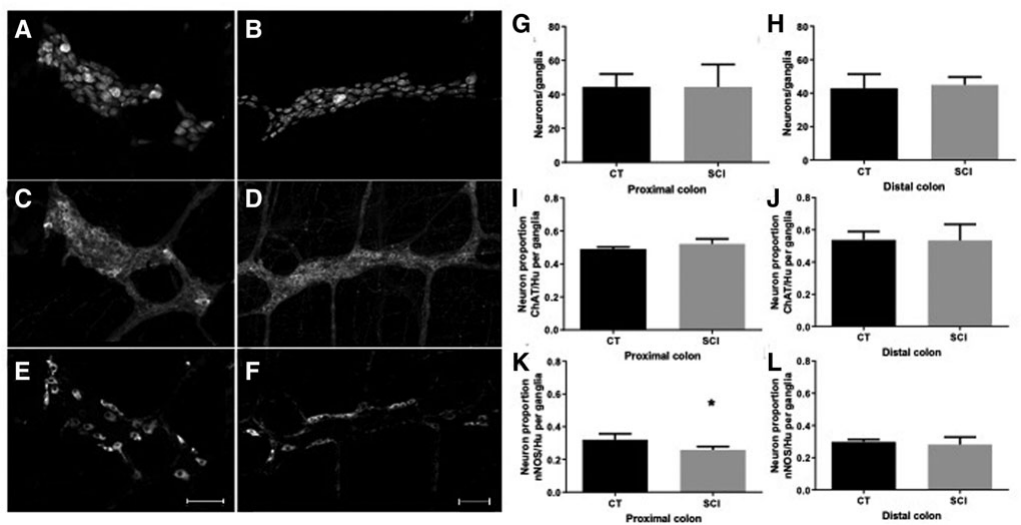
Impact of spinal cord injury (SCI) on neurochemical coding of myenteric neurons in the proximal and distal colon. Triple immunochemical staining of whole-mount preparations of myenteric plexus and longitudinal muscle layer using antibodies against Hu **(A,B)**, choline acetyltransferase (ChAT; **C,D**) and neuronal nitric oxide synthase (nNOS; **E,F**) in the proximal colon. SCI (B and C) did not modify the number of neurons per ganglia as compared with controls (CT) (sham enemas) in the proximal and distal colon (**G** and **H**, respectively). SCI did not change the proportion of ChAT-immunoreactive (IR) neurons per ganglia compared with CT in the proximal and distal colon (D and F). Finally, SCI reduced the proportion of nNOS-IR neurons per ganglia in the proximal and distal colon compared with CT (G and **I**); (*p < 0.05) (scale bar = 100 μm).

In this part of the study we aimed to determine whether SCI induced changes in the neurochemical phenotype of myenteric neurons in proximal and distal colon (Fig. 3).

First, using the general neuronal marker HU, we showed that the number of myenteric neurons per ganglion was comparable in the proximal colon between SCI animals (Fig. 3B) and CT (Fig. 3A) (44.5 ± 7.5 vs. 44.4 ± 13.2 Hu-IR neurons/ganglion, respectively; *p* = 0.84) (Fig. 3G). The number of myenteric neurons per ganglion was also comparable in the distal colon between SCI animals and CT (44.9 ± 4.7 vs. 42.9 ± 8.4 Hu-IR neurons/ganglion, respectively; *p* = 0.84) (Fig. 3H).

Next, we determined the proportion of ChAT-IR and nNOS-IR neurons in the myenteric plexus of SCI and CT animals. In the proximal colon, the proportion of ChAT/Hu myenteric neurons per ganglion tended to be increased in SCI (Fig. 3D) compared with CT (Fig. 3C) (52.1% and 49.0%, respectively; *p* = 0.09) (Fig. 3I). The proportion of nNOS/Hu neurons was significantly reduced in SCI animals (Fig. 3F) compared with CT (Fig. 3E) (25.8% and 32.1%, respectively; *p* = 0.03) (Fig. 3K).

In the distal colon, the proportion of ChAT/Hu myenteric neurons per ganglion was comparable between SCI and CT (53.3% and 53.8%, respectively; *p* = 0.99) (Fig. 3J). In addition, the proportion of nNOS/Hu neurons was also comparable between SCI animals and CT (28.1% and 29.8%, respectively; *p* = 0.55) (Fig. 3L).

### Impact of SCI on the colonic concentration of acetylcholine

**FIG. 4.**
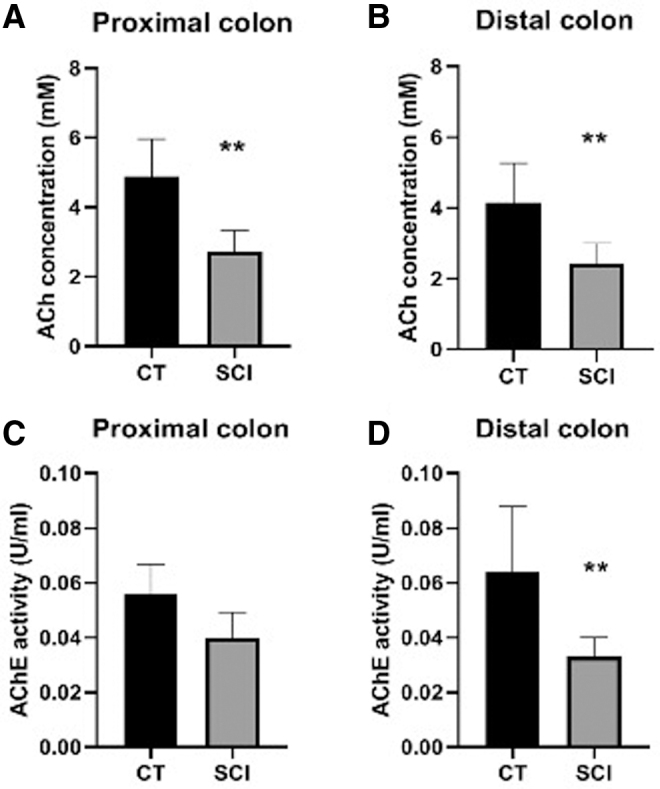
Impact of spinal cord injury (SCI) upon acetycholine (Ach) concentration and acetylcholinesterase (AchE) activity on the proximal and distal colon. Concentration of Ach was significantly reduced in both the proximal **(A)** and distal **(B)** colon compared with control (CT) (*p* = 0.0008 for both). In addition, AchE activity was decreased in the distal colon **(C)** compared with CT (*p* = 0.0001).

Although the proportion of ChAT/Hu myenteric neurons per ganglion was comparable between the SCI and CT animals, we aimed to determine whether acetylcholine levels in proximal and distal colon were affected or not by SCI. Using an acetylcholine/acetylcholinesterase assay, we measured in the proximal and distal colon of SCI rats, a significantly lower acetylcholine concentration than in CT rats (*p* = 0.008 for both) (Fig. 4A,B). In addition, the acetylcholinesterase activity was lower in SCI in the distal colon (*p* = 0.001) (Fig. 4D) and tended to be lower in the proximal colon (*p* = 0.06) (Fig. 4C) compared with CT.

### Impact of the spinal cord injury on the colonic muscle response

**FIG. 5.**
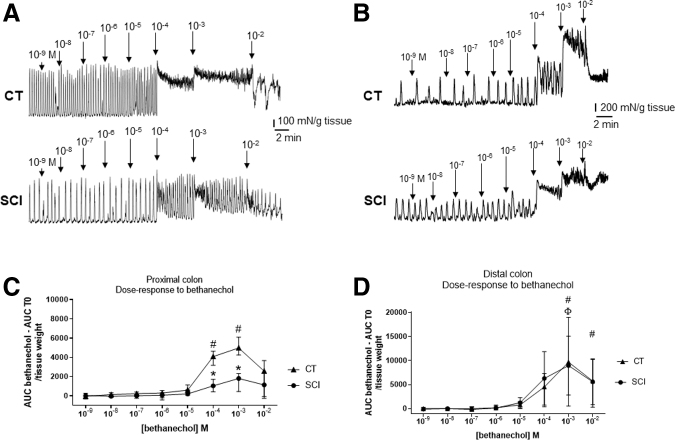
Example of evolution of spontaneous contractions under increasing concentrations of bethanechol in proximal **(A)** and distal **(B)** colon, in the two groups, spinal cord injury (SCI) and control (CT). Evolution of the area under the curve (AUC) at the different concentrations of bethanechol, in the proximal colon of the two groups. AUC T0 = area under the initial curve, before drug application (# and Φ for response significantly different from the initial AUC and * for significantly different response between SCI and CT groups) **(C)**. Evolution of the AUC at the different concentrations of bethanechol, in the distal colon of the two groups. AUC T0 = area under the initial curve, before drug application (#and Φ for response significantly different from the initial AUC and * for significantly different response between SCI and CT groups) **(D)**. Example of dose-response to bethanechol in proximal colon of CT and SCI rats.

Finally, we aimed to determine whether the sensitivity of the muscle response to muscarinic receptor agonist was altered in SCI compared with CT. We stimulated *ex vivo* the digestive tract segments with increasing doses of bethanechol in the proximal and distal colon (Fig. 5A,B).

In the proximal colon, bethanechol induced a significant increase in the contractile response for the concentrations of 10^−4^ and 10^−3^M compared with CT (i.e., absence of bethanechol; Friedman test: *p* < 0.001), in CT (Dunn test, *p* < 0.05 #) but not in SCI rats. In addition, the AUC at bethanechol 10^−4^ and 10^−3^ M concentrations was lower in the SCI rats compared with the CT rats (two-way ANOVA repeated measure [RM], *p* < 0.0001, Bonferroni test, *p* < 0.05*) (Fig. 5C).

In the distal colon, bethanechol induced a significant increase in the contractile response for the concentrations of 10^−3^ and 10^−2^M compared with CT (i.e., absence of bethanechol) (Friedman test: *p* < 0.0001) in CT (Dunn test, *p* < 0.05 #) and in the SCI group (Dunn test, *p* < 0.05*). In addition, the AUC at bethanechol 10^−4^ and 10^−3^ M concentrations was similar in the SCI and CT rats (two-way ANOVA MR, *p* > 0.05, Bonferroni's test, *p* > 0.05) (Fig. 5D).

### Impact of spinal cord injury on colonic inflammation

In the last part of the study, we aimed to determine whether changes in ENS and colonic motor responses were associated with changes in inflammatory cytokines in the different colonic segments.

**FIG. 6.**
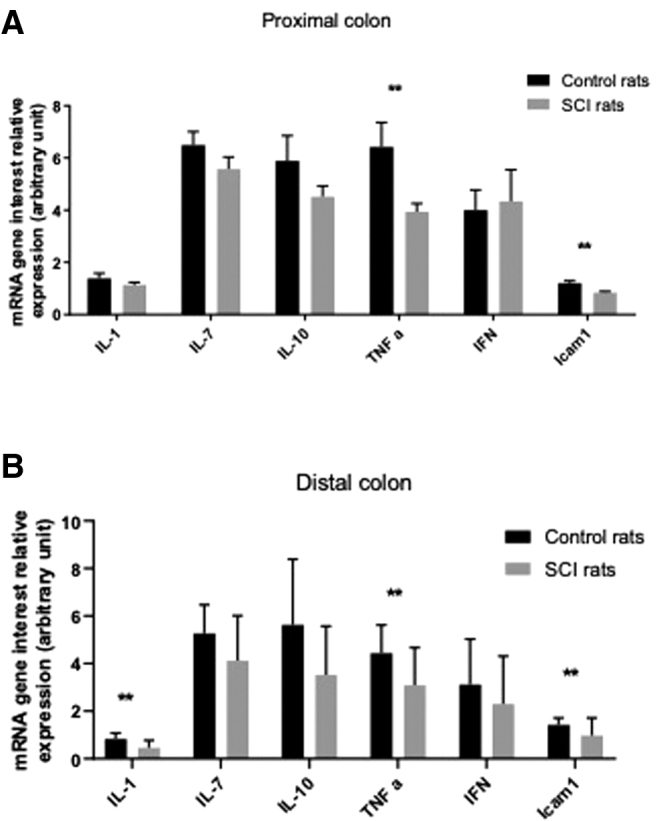
Expression of interleukin (IL)-1, IL-7, IL-10, tumor necrosis factor alpha (TNF-α), interferon (IFN), and intercellular adhesion molecule (ICAM) messenger RNA (mRNA) in proximal **(A)** and distal colon **(B)**.

In particular, we found that mRNA expression of TNF-α and ICAM-1 was significantly reduced in SCI rats compared with CT (*p* = 0.005 and *p* = 0.002, respectively) in the proximal colon (Fig. 6A). No changes in IL-1, IL-7, IL-10, or interferon gamma (IFNγ) occurred. In the distal colon, mRNA expression of TNF-α and ICAM-1, but also IL-1, was significantly lower in SCI rats compared with CT (*p* = 0.005, *p* = 0.007, and *p* = 0.007, respectively), whereas no change in IL-7, IL-10, or IFNγ was measured (Fig. 6B).

## Discussion

This study was designed to characterize the impact of SCI on the potential remodeling of the ENS and its functional consequences on *ex vivo* colonic motor function in rats. Using a rat model of chronic thoracic SCI, we described a region-specific neurochemical remodeling of the ENS characterized by a significant decrease in the proportion of nitrergic neurons in the proximal but not distal colon compared with CT. Further, we also showed a significant reduction in the tissue acetycholine concentration (but not in the proportion of ChAT neurons) in the proximal but not distal colon. In addition, *ex vivo*, we showed a statistical trend toward changes in contractile response induced by ENS activation affecting the proximal but not distal colon and characterized by a tendency to reduce its cholinergic but not nitrergic component. Finally, we showed that the sensitivity of the muscle to bethanechol was reduced in spinalized rats compared with CT. This work highlights a remodeling of the ENS and the muscle of the proximal colon versus distal colon that may contribute to the digestive disorders associated with SCI.

One of the main results of this study is the selective involvement (contractile response, phenotypic modification) of the proximal colon after spinalization compared with the distal colon. Our results are different from those of Joo and colleagues.^20^ Indeed, they showed that the contractility was significantly higher in the proximal colon of the SCI rats than that in the proximal colon of the CT rats.

One possible hypothesis to explain these differences is that changes reported occurred after 1 week post-SCI, whereas changes reported in our model occurred after 4 weeks post-SCI. The mechanisms underlying the region-specific modification of the proximal versus distal colon after spinalization could be related to the anatomical properties of the innervation of the colon by the extrinsic nervous system. Indeed, in our model, rats were spinalized into T8, with a metameric spinal cord transection and upper motor neuron lesion (presence of all sublesional reflexes). This leaves intact the sacral parasympathetic center (from L6 to S1 in rats) and its afferences toward the distal colon. The thoracolumbal sympathetic center is much closer to the lesion (in rats theoretically from T10 to L2, as in humans). Further, Ferrero and associates^21^ showed that spinalization of rats above T7 was a safer way to have a rapid recovery of bladder reflexes, that is, a lesion below this level would probably reach at least the thoracolumbar sympathetic center. Because the extrinsic sympathetic innervation comes from the thoracolumbal center in the proximal colon, it might explain that the proximal colon is more affected than the distal part of the colon.

A major finding of our study was the significant reduction in acetylcholine concentration in the proximal but not distal colon. Although no change in the proportion of cholinergic neurons was observed, one cannot exclude that a reduction in neuronal ChAT concentration (to a level still allowing detection by immunhistochemical methods) or a reduction in ChAT activity could occur. Conversely, besides neurons, ChAT expression has also been reported in other cell types such as epithelial and immune cells, and reduction in tissue acetylcholine concentration could therefore reflect changes in these cells.^22^ Besides reduced acetylcholine expression in the proximal colon, we also observed a reduced sensitivity of the colonic muscle to the muscarinic agonist bethanechol. These modifications could also be due to altered expression of muscarinic receptors present on smooth muscle cells or downstream alterations of signaling pathways and effectors activated by these receptors.^23^ Surprisingly, this significant decrease in tissue acetylcholine concentration and the decrease in muscle sensitivity to acetylcholine did not lead to change in EFS-induced contractile response in SCI animal compared with CT. This suggests that SCI induces a remodeling in the ENS of other excitatory mediators such as substance P. This putative remodeling could counteract the functional impairment in the cholinergic component following EFS.

Interestingly, the proportion of nitrergic neurons in the ENS is decreased after SCI. These results are in agreement with a previous study that also found a decrease in IR for nNOS at 48 h and 7 days of spinalization in the stomach and small intestine,^14^ but no data on the colon were provided in the study. In our study, we reported no change in the nitrergic component of the EFS-induced contractile response. This could result from the fact that we studied neuromuscular transmission in the longitudinal muscle that has been shown to the mainly innervated by cholinergic neurons.^13^ *In vivo*, the changes could also contribute to digestive tract disorders in SCI, as various studies have shown that a decrease in the expression of nNOS slows intestinal transit.^24^

Among factors explaining changes in the phenotype of the ENS-induced SCI, various hypotheses can be suggested. First, spinalization could result in a loss of innervation of enteric neurons by extrinsic fibers that could in turn impact upon modulation in enteric neuronal activity. The latter could contribute to changes in neurotransmitter expression of enteric neurons, as neuronal activity was shown to increase neurotransmitter gene expression in the ENS.^25^ Other factors such as the intestinal microbiota may also be involved. Indeed, SCI has been shown to induce changes in microbiota composition and metabolites expression.^26^ These changes in bacterial composition could be a consequence of changes in digestive function (induced by SCI). Conversely, microbiota and bacterial metabolites can modulate the phenotype of the ENS, in particular nitrergic and/or cholinergic neurons.^27,28^ In particular, it has been shown that expression of ChAT/acetylcholine is modulated by bacterial metabolites such as butyrate.^29^

In conclusion, our study highlights that SCI leads to a remodeling of the ENS and functional consequences that may be consistent with clinical findings. The identification of these mechanisms may lead to improved management of digestive dysfunctions in SCI. Further, studies are needed to confirm these results in animals and then in humans.

## Abbreviations Used

ANOVA: analysis of variance
AUC: area under the curve
ChAT: choline acetyltransferase
CT: control
Cy3: carboxymethylindocyanine
Cy5: 7-amino-4-indodicarbocyanin
EFS: electrical field stimulation
ENS: enteric nervous system
FICT: fluorescein isothiocyanate
ICAM-1: intercellular adhesion molecule-1
IL: interleukin
IR: immunoreactive
L-NAME: *N*-nitro-L-arginine methyl ester
mRNA: messenger RNA
nNOS: neuronal nitric oxide synthase
NOS: nitric oxide synthase
PBS: phosphate-buffered saline
PFA: paraformaldehyde
RIPA: radioimmunoprecipitation assay
SCI: spinal cord injury

## References

[1] Brading, AF., and Ramalingam, T. (2006). Mechanisms controlling normal defecation and the potential effects of spinal cord injury. Prog. Brain Res. 152, 345–358 16198712 10.1016/S0079-6123(05)52023-5

[2] Chung, E.A.L., and Emmanuel, A.V. (2006). Gastrointestinal symptoms related to autonomic dysfunction following spinal cord injury. Prog. Brain Res. 152, 317–333 16198710 10.1016/S0079-6123(05)52021-1

[3] Enck, P., Greving, I., Klosterhalfen, S., and Wietek, B. (2006). Upper and lower gastrointestinal motor and sensory dysfunction after human spinal cord injury. Prog. Brain Res. 152, 373–384 16198714 10.1016/S0079-6123(05)52025-9

[4] Coggrave, M., and Norton, C. (2013) Management of faecal incontinence and constipation in adults with central neurological diseases. Cochrane Database Syst. Rev. 18, CD002115 10.1002/14651858.CD002115.pub4 24347087

[5] Burns, AS., St.-Germain, D., Connolly, M., Delparte, J.J., Guindon, A., and Hitzig, S.L. (2015). Phenomenological study of neurogenic bowel from the perspective of individuals living with spinal cord injury. Arch. Phys. Med. Rehabil. 96, 49–55 25172370 10.1016/j.apmr.2014.07.417

[6] Hanson, R.W., and Franklin, M.R.Sexual loss in relation to other functional losses for spinal cord injured males. (1976). Arch. Phys. Med. Rehabil. 57, 291–293 1275682

[7] Anderson, KD.Targeting recovery: priorities of the spinal cord-injured population. (2004). J. Neurotrauma 21, 1371–1383 10.1089/neu.2004.21.1371 15672628

[8] Bloemen-Vrencken, J.H.A., de Witte, L.P., and Post, M.W.M. Follow-up care for persons with spinal cord injury living in the community: a systematic review of interventions and their evaluation. (2005). Spinal Cord 43, 462–475 15838530 10.1038/sj.sc.3101750

[9] Snoek, G.J., IJzerman, M.J., Hermens, H.J., Maxwell, D., and Biering-Sorensen, F. Survey of the needs of patients with spinal cord injury: impact and priority for improvement in hand function in tetraplegics. (2004). Spinal Cord 42, 526–532 10.1038/sj.sc.3101638 15224087

[10] Ditunno, P.L., Patrick, M., Stineman, M., and Ditunno, J.F. Who wants to walk? Preferences for recovery after SCI: a longitudinal and cross-sectional study. (2008). Spinal Cord 46, 500–506 18209742 10.1038/sj.sc.3102172

[11] Simpson, L.A., Eng, J.J., Hsieh, J.T.C., Wolfe, D.L., and Spinal Cord Injury Rehabilitation Evidence Scire Research Team. The health and life priorities of individuals with spinal cord injury: a systematic review. (2012). J. Neurotrauma 29,1548–1555 10.1089/neu.2011.2226 PMC3501530 22320160

[12] Holmes, G.M., Hudson, T.R., and Filart, R. (2017). Neurogastroenterology in spinal cord dysfunction, in: *Neurological Aspects of Spinal Cord Injury*. W. Weidner, R. Rupp, and K. Tansey (eds). Springer, pps. 397–437

[13] Schemann, M., and Neunlist, M. The human enteric nervous system. (2004). Neurogastroenterol. Motil. 16, 55–59 10.1111/j.1743-3150.2004.00476.x 15066006

[14] Kabatas, S., Yu, D., He, X.D., Thatte, H.S., Benedict, D., and Hepgul, K.T. (2008). Neural and anatomical abnormalities of the gastrointestinal system resulting from contusion spinal cord injury. Neuroscience 154,1627–1638 18556138 10.1016/j.neuroscience.2008.04.071

[15] Joo, M.C., Kim, Y.S., Choi, E.S., Oh, J.T., Park, H.J., and Lee, M.Y. (2011). Changes in the muscarinic receptors on the colonic smooth muscles of rats with spinal cord injury. Ann. Rehabil. Med. 35, 589–598 22506180 10.5535/arm.2011.35.5.589 PMC3309258

[16] White, AR., and Holmes, GM. Anatomical and Functional Changes to the Colonic Neuromuscular Compartment after Experimental Spinal Cord Injury. (2018). J. Neurotrauma 35, 1079–1090 10.1089/neu.2017.5369 PMC5908428 29205096

[17] Liu, C.-W., Huang, C.-C., Chen, C.-H., Yang, Y.-H., Chen, T.-W., and Huang, M.-H. Prediction of severe neurogenic bowel dysfunction in persons with spinal cord injury. (2010). Spinal Cord 48, 554–559 20065986 10.1038/sc.2009.181

[18] Vallès, M., Vidal, J., Clavé, P., and Mearin, F. (2006). Bowel dysfunction in patients with motor complete spinal cord injury: clinical, neurological, and pathophysiological associations. Am. J. Gastroenterol.101, 2290–2299 10.1111/j.1572-0241.2006.00729.x 17032195

[19] Behr-Roussel, D., Oger, S., Caisey, S., Sandner, P., Bernabé, J., Alexandre, L., and Giuliano, F. (2011). Vardenafil decreases bladder afferent nerve activity in unanesthetized, decerebrate, spinal cord-injured rats. Eur. Urol. 59, 272–279 21036463 10.1016/j.eururo.2010.10.037

[20] Joo, M.C., Kim, Y.S., Choi, E.S., Oh, J.T., Park, H.J., and Lee, M.Y.Changes in the muscarinic receptors on the colonic smooth muscles of rats with spinal cord injury. (2011). Ann. Rehabil. Med. 35, 589–598 10.5535/arm.2011.35.5.589 PMC3309258 22506180

[21] Ferrero, S.L., Brady, T.D., Dugan, V.P., Armstrong, J.E., Hubscher, C.H., and Johnson, R.D.Effects of lateral funiculus sparing, spinal lesion level, and gender on recovery of bladder voiding reflexes and hematuria in rats. (2015). J. Neurotrauma 32, 200–208 10.1089/neu.2013.3247 PMC4298755 25137571

[22] Takahashi, K., Kitamura, N., Suzuki, Y., Yamanaka, Y., Shinohara, H., and Shibuya, I. (2015). Activation of muscarinic acetylcholine receptors elevates intracellular Ca(2+) concentrations in accessory lobe neurons of the chick. J. Comp. Physiol. A. Neuroethol. Sens. Neural Behav. Physiol. 201, 385–394 25481714 10.1007/s00359-014-0971-6

[23] Gerthoffer, W.T. (2005). Signal-transduction pathways that regulate visceral smooth muscle function. III. Coupling of muscarinic receptors to signaling kinases and effector proteins in gastrointestinal smooth muscles. Am. J. Physiol. Gastrointest. Liver Physiol. 288, G849–G853 15826932 10.1152/ajpgi.00530.2004

[24] Dickson, E.J., Heredia, D.J., McCann, C.J., Hennig, G.W., and Smith, T.K. (2010). The mechanisms underlying the generation of the colonic migrating motor complex in both wild-type and nNOS knockout mice. Am. J. Physiol. Gastrointest. Liver. Physiol. 298, G222–G232 19959818 10.1152/ajpgi.00399.2009 PMC2822500

[25] Chevalier, J., Derkinderen, P., Gomes, P., Thinard, R., Naveilhan, P., and Vanden Berghe, P. (2008). Activity-dependent regulation of tyrosine hydroxylase expression in the enteric nervous system. J. Physiol. (Lond.) 586, 1963–1975 18258664 10.1113/jphysiol.2007.149815 PMC2375718

[26] Gungor, B., Adiguzel, E., Gursel, I., Yilmaz, B., and Gursel, M. (2016). Intestinal microbiota in patients with spinal cord injury. PLoS One 11, e0145878 26752409 10.1371/journal.pone.0145878 PMC4709077

[27] Anitha, M., Reichardt, F., Tabatabavakili, S., Nezami, B.G., Chassaing, B., and Mwangi, S. Intestinal dysbiosis contributes to the delayed gastrointestinal transit in high-fat diet fed mice. (2016). Cell Mol. Gastroenterol. Hepatol. 2, 328–339 10.1016/j.jcmgh.2015.12.008 PMC4945127 27446985

[28] Hyland, N.P., and Cryan, J.F.Microbe-host interactions: influence of the gut microbiota on the enteric nervous system. (2016). Dev. Biol. 417, 182–187 10.1016/j.ydbio.2016.06.027 27343895

[29] Soret, R., Chevalier, J., De Coppet, P., Poupeau, G., Derkinderen, P., and Segain, J.P. (2010). Short-chain fatty acids regulate the enteric neurons and control gastrointestinal motility in rats. Gastroenterology. 138,1772–1782 20152836 10.1053/j.gastro.2010.01.053

